# Heat tolerance during embryonic development has not diverged among populations of a widespread species (*Sceloporus undulatus*)

**DOI:** 10.1093/conphys/cot018

**Published:** 2013-06-11

**Authors:** Michael J. Angilletta, Maximilian H. Zelic, Gregory J. Adrian, Alex M. Hurliman, Colton D. Smith

**Affiliations:** School of Life Sciences, Arizona State University, Tempe, AZ 85287, USA

**Keywords:** Critical thermal maximum, embryo, heart rate, survival, temperature, thermal tolerance

## Abstract

The frequency and magnitude of heat waves have increased in recent decades, imposing additional stresses on organisms in extreme environments. Most reptilian embryos are regularly exposed to thermal stress because they develop in shallow, warm soils for weeks to months. We studied cardiac performance during warming to infer lethal temperatures for embryonic lizards in the *Sceloporus undulatus* complex. Embryos from four populations throughout the geographical range (New Jersey, South Carolina, Colorado, and Arizona) were warmed at a rate observed in natural nests. Embryos from all populations exhibited a similar pattern of thermal sensitivity, as follows: heart rate rose between 34 and 41°C, remained stable between 41 and 44°C, and dropped sharply between 44 and 47°C. No embryos recovered from cardiac arrest, indicating that the upper lethal temperature was ≤47°C. Despite the putative selective pressures, the thermal limit to cardiac performance seems to have been conserved during the evolution of this species.

## Abstract (precis)

Animals that develop in shallow soils are susceptible to lethal temperatures during heat waves. We found that developing lizards from four populations entered cardiac arrest at temperatures above 46°C. Since temperatures of natural nests can presently exceed this limit, global warming would further reduce recruitment of young.

## Introduction

In the last decade, researchers have increasingly used organismal tolerances to predict impacts of climate change on population dynamics or geographical distributions (Braune *et al.*, 2008; Buckley *et al.*, 2010; Kearney, 2011; Molnar *et al.*, 2013). The development and application of their models depend on empirical estimates of tolerance during realistic changes in environmental conditions. Many, if not most, organisms experience rapid and wide fluctuations in temperature within hours to days. Indeed, heat waves have become common in recent decades (Hansen *et al.*, 2012) and should occur even more frequently in the coming decades (IPCC, 2007). When the effects of such acute stresses were unknown, studies of chronic stresses were used to parameterize models (Crozier and Dwyer, 2006; Deutsch *et al.*, 2008). This approach must bias predictions, because organisms thrive during periodic exposure to temperatures that prove lethal during chronic exposure (Siddiqui and Barlow, 1972, 1973; Siddiqui *et al.*, 1973). Therefore, organismal responses to acute stresses must be documented to infer the biological impacts of climate change (e.g. see Huey *et al.*, 2009).

Animals should be particularly vulnerable to sudden warming at the embryonic stage. Embryos, being relatively small and immobile, have limited capacities to thermoregulate (but see Du *et al.*, 2011). Despite the danger that acute thermal stress should pose to embryos, we know virtually nothing about the impacts of this stress on embryonic performance. Studies of natural nests suggest that thermal fluctuations have little direct impact on embryonic performance (Cagle *et al.*, 1993; Thompson *et al.*, 1996; Shine *et al.*, 1997); however, these studies cannot reveal the impacts of temperatures that exceed the current range. Although many researchers have manipulated fluctuations in temperature (Qualls and Shine, 1998; Ashmore and Janzen, 2003; Shine, 2004; Mullins and Janzen, 2006; Oufiero and Angilletta, 2006; Du and Shine, 2010; Warner *et al.*, 2010; Warner and Shine, 2011), these experiments were not designed to stress embryos. Consequently, embryos were not exposed to temperatures that approached or exceeded their thermal limit of performance.

Admittedly, quantifying the impacts of thermal stress on embryos remains difficult because no standard assay exists. For other life stages, researchers can choose from several assays to gauge whether animals can tolerate severe and sudden thermal stress (see reviews by Lutterschmidt and Hutchison, 1997; Hoffmann *et al.*, 2003; Angilletta, 2009). These assays focus on the maximal temperature at which an animal can move or respond to mechanical stimuli, usually referred to as the critical thermal maximum (Cowles and Bogert, 1944) or knockdown temperature (Huey *et al.*, 1992). By warming an animal over the course of minutes to hours, a researcher can pinpoint the temperature at which the animal loses some ability. For example, isopods lost their ability to roll over at temperatures around 37°C (Castaneda *et al.*, 2004), and flies lost their ability to fly between 35 and 42°C (Gilchrist and Huey, 1999). Likewise, various species of fish (Beitinger *et al.*, 2000) and reptiles (Licht *et al.*, 1966; Winne and Keck, 2005) ceased to move at specific temperatures. However, these assays target behaviours that one cannot observe directly for embryos.

Fortunately, methodological advances enable one to record heart rates of amniotic embryos without disturbing their growth and development (Lierz *et al.*, 2006; Du and Shine, 2008). Changes in heart rate reveal physiological responses to warming. As an organism warms, its heart initially beats faster (Du *et al.*, 2010c). This acceleration enhances the distribution of oxygen and nutrients to meet the greater metabolic demands of a warm body (Du *et al.*, 2009, 2010b). But the beating of the heart cannot accelerate indefinitely. At some temperature, the heart will fail to beat faster or even fail to beat at all. Such changes in cardiac performance have been used to infer thermal limits in adult ectotherms (Stillman and Somero, 1996).

**Figure 1:**
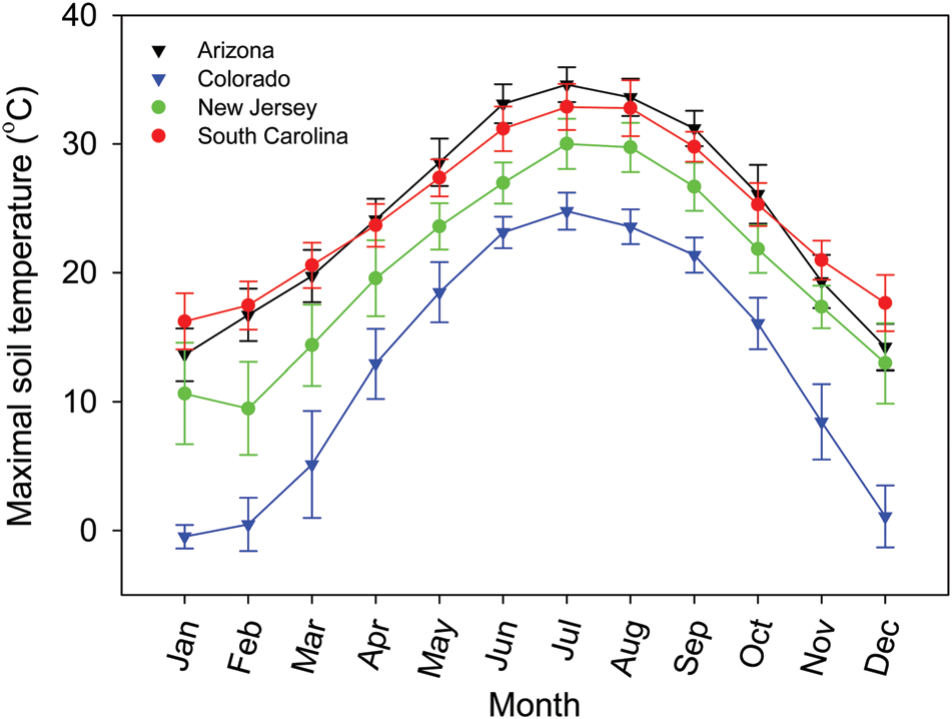
Soils in Arizona and South Carolina are warmer than those in Colorado and New Jersey. Maximal soil temperatures at a depth of 5 cm at localities closest to our populations: AZ, 33.2° N, 110.0° W; NJ, 39.8° N, 74.7° W; SC, 33.6° N, 81.9° W; and CO, 37.0° N, 105.6° W. Each datum is the mean and standard deviation of monthly maxima between 1979 and 2010, averaged over a grid of 0.312° × 0.312° (National Center for Atmospheric Research, Boulder, CO, USA).

We used changes in heart rate to quantify the heat tolerance of embryonic lizards in the *Sceloporus undulatus* complex. This paraphyletic biological species comprises four major clades that collectively cover much of the USA (Leaché and Reeder, 2002; Leaché, 2009). Both morphology and life history vary within clades according to environmental temperatures (Angilletta and Sears, 2004; Angilletta *et al.*, 2004, 2006). However, the heat tolerance of adults seems highly conserved throughout the range, possibly because of effective thermoregulation (Ehrenberger, 2010). As embryos cannot behaviourally thermoregulate to the same degree that adults can, we expected their heat tolerances to reflect local climates (Fig. 1); specifically, we expected embryos from warm, southern regions to tolerate higher temperatures than do embryos from cool, northern regions. To test this prediction, we compared the thermal sensitivity and critical thermal maximum of cardiac performance between northern and southern populations from two clades.

## Materials and methods

### Ethics statement

This study was conducted according to recommendations in the Guidelines for Use of Live Amphibians and Reptiles in Field and Laboratory Research of the American Society of Ichthyologists and Herpetologists. All procedures were approved by the Animal Care and Use Committee of Arizona State University (protocol no. 11-1161).

### Collection and husbandry

Although lizards throughout the range of *S. undulatus* reproduce at different frequencies, lizards in all populations produce eggs between May and July. Therefore, we collected gravid females in May 2012. These lizards came from the following four localities: Atlantic County, NJ; Edgefield County, SC; Costilla County, CO; and Gila County, AZ. Lizards were transported to Arizona State University, where they were housed in plastic terraria with a substrate of moist sand. Terraria were kept in a controlled environment, with a 12 h light–12 h dark cycle, and an ambient temperature of 33°C during photophase and 20°C during scotophase. Water was available at all times, and crickets were provided daily.

### Acquisition and care of eggs

Females either laid eggs naturally or were induced with an injection of oxytocin, as described by Storm and Angilletta (2007). Freshly laid eggs were weighed and placed in plastic containers for incubation. Each container contained ∼400 g of sand mixed with 4 g of water, resulting in a water potential of −10 kPa (Oufiero and Angilletta, 2006).

All containers were kept in a programmable incubator (model DR-36VL; Percival Scientific). The incubator maintained a daily cycle of temperature, ranging from 20.4 to 34.7°C, and a relative humidity of 85%. The thermal cycle was based on the temperatures of nests constructed by females in artificial thermal gradients and natural environments (Warner and Andrews, 2002; Angilletta *et al.*, 2009). Water that evaporated from the containers was replaced weekly. Although this procedure permitted small changes in water potential, eggs incubated in these conditions absorb water at a relatively constant rate throughout incubation (Angilletta *et al.*, 2000). One egg from each population was incubated in each container to prevent covariation between environmental and genetic factors.

### Thermal sensitivity of heart rate

We measured the effect of temperature on the heart rates of embryos throughout development. Heart rates were measured using a commercially available system of infrared sensors (Buddy Egg Monitor; Avitronics, Truro, UK); heart rates estimated by this method compare favourably with those estimated by other methods (Lierz *et al.*, 2006). Heart rates of lizard embryos became detectable about a week after oviposition. From this point onwards, we recorded heart rates at 24 and 34°C for one embryo of each clutch (23, 17, 7, and 7 clutches from NJ, SC, CO, and AZ, respectively). Measurements were made weekly, at the point in the daily cycle when eggs would normally experience these temperatures (10.00 and 14.00 h for 24 and 34°C, respectively). Immediately before each measurement, eggs were temporarily moved to containers of moist sand, similar to the incubation containers. Containers were placed in an incubator set at either 24 or 34°C. Eggs were removed from the incubator one at a time, enabling us to transfer each egg quickly between the incubator and the heart rate monitor. Heart rate was recorded within 30 s to minimize the cooling of embryos during measurements. After measurements at each temperature, the eggs were returned to their incubation containers and placed in the incubator that maintained their daily cycle of temperature.

Using an information-theoretical approach (Burnham and Anderson, 2002), we identified the best statistical model of heart rate. General linear modelling was used to estimate the effects of temperature, initial egg mass, stage of incubation (estimated as days since oviposition), and population. To avoid pseudoreplication, we also included the identity of each egg as a random factor (Zuur *et al.*, 2009). Initially, we modelled all main effects and interactions. Then, following Crawley (2007), we dropped terms from the maximal model and used Akaike's information criterion to confirm the improved fit of the simplified model. We removed terms from the highest order to the lowest order until the model with the lowest Akaike's information criterion was obtained. All models were fitted using the *nlme* library (Pinheiro *et al.*, 2013) of the R Statistical Package (R Development Core Team, 2011).

### Thermal limits of heart rate

We estimated the thermal limit of cardiac performance by monitoring the heart rates of embryos during continuous warming. Measurements began at 14.00 h when eggs would normally experience 34°C (see ‘*Acquisition and care of eggs*’). An egg from each clutch was randomly assigned to a warming group and a control group (19, 11, 5, and 7 clutches from NJ, SC, CO, and AZ, respectively). Eggs of the former group were warmed by 3°C h^−1^ from 34°C to their upper lethal limits, whereas eggs of the latter group were handled in a similar manner while remaining at their usual cycle of temperature. The rate of warming lies within the range of rates reported for natural nests (Angilletta *et al.*, 2009). To control warming while recording heart rates at specific intervals, we warmed only two eggs per trial. The two control eggs in each trial were from the same clutches.

We took precautions to ensure that eggs warmed precisely and remained at the target temperatures during measurements of heart rate. At the start of each trial, eggs were weighed to the nearest 0.01 mg. Each egg was positioned in a padded dish and sealed in a glass jar (125 ml); the jar contained ∼1 ml of distilled water to prevent the egg from desiccating through evaporation. A thermocouple was inserted through the lid of the jar to monitor the air temperature inside. The jar was then submerged in a water bath, whose temperature was regulated by a programmable circulator (Proline 855C; LAUDA-Brinkmann, LP). Initially, the temperature of the bath was set to 34°C. A few minutes after the temperature inside the jar reached 34°C, the jar was transferred to a small container of water at the same temperature as the bath. This container was carried into an environmental chamber, which was set at the same temperature as the bath. Inside the chamber, the egg was quickly removed from the jar and placed inside a heart-rate monitor. Although the heart rate was recorded manually, we also used a video camera to record the digital panel of the heart-rate monitor for ∼30 s; this recording enabled us to confirm any values that seemed unusual during data analysis. Immediately after this recording, the egg was placed in its jar and returned to the water bath. The temperature of the bath was incremented by 0.5°C, and the entire procedure was repeated. For each egg, the trial ended when we were unable to obtain a heart rate for two consecutive intervals. Eggs in the control group remained at their usual cycle of temperature; each time that heart rates of the warming embryos were measured, the control eggs were placed in a small box for the same duration to simulate the handling that occurs during measurements of heart rate. At the end of the trial, eggs were reweighed to estimate water loss during the experiment.

We used general additive mixed modelling to compare thermal sensitivities of heart rate among populations. An additive model permitted a non-linear response to temperature, without requiring us to specify a function (Zuur *et al.*, 2009). Consequently, we preferred this approach to one that assumed an exponential, an asymptotic, or a piecewise function (e.g. Arrhenius breakpoints). Potential explanatory variables included temperature, population, and stage of incubation. To avoid pseudoreplication, the identity of each embryo was included as a random factor. For each combination of factors, we fitted a model with a fixed error term and a model in which the error in heart rate increased with increasing temperature.

Given that non-linear models were unable to fit the sharp drop in heart rate at high temperatures, we used the raw data to estimate the critical thermal maximum of heart rate, i.e. the temperature at which heart rate dropped to zero. The method of general least squares was used to model the critical thermal maximum as a function of population, egg mass, and stage of incubation. A separate error term was estimated for each population, because the variances appeared to differ. Analyses were performed with the *mgcv* (Wood, 2004) and *nlme* (Pinheiro *et al.*, 2013) libraries of R. The most likely model was selected on the basis of Akaike's information criterion.

### Phenotypes of hatchlings

After 45 days of incubation, eggs were checked daily for signs of hatching. Hatchlings were weighed to the nearest 0.01 g and measured to the nearest millimetre (snout-to-vent length). Descriptive statistics are reported as means ± 95% confidence interval.

## Results

### Thermal sensitivity of cardiac performance

**Figure 2:**
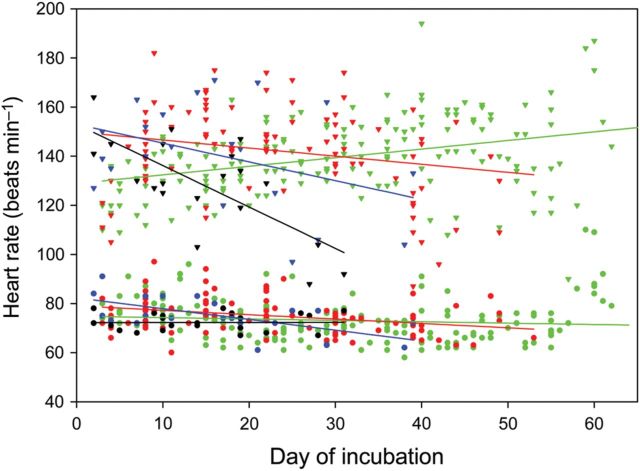
Mean heart rates of embryos at 24 (circles) and 34°C (triangles) changed slightly throughout development. Data are for lizards from Arizona (black symbols), Colorado (blue symbols), New Jersey (green symbols), and South Carolina (red symbols). Lines represent the relationships estimated from the most likely statistical model.

The most likely model of heart rate included many sources of variation, including egg mass at oviposition, population of origin, stage of incubation, and some interactions among these variables (Table 1). Nevertheless, body temperature affected the heart rate of embryos far more than any other factor. At all stages of incubation, heart rate at 34°C exceeded that at 24°C by at least 70%. For most populations, mean heart rate remained stable or decreased slightly throughout incubation (Fig. 2).

**Table 1:** Inferential statistics for the most likely model of heart rate at tolerable temperatures

Effect	Effect d.f.	Error d.f.	*F*	*P*-value
Intercept	1	609	242.93	<0.0001
Mass at oviposition (g)	1	609	0.68	0.4096
Stage of incubation (days)	1	609	5.91	0.0153
Population (AZ, CO, NJ, SC)	3	609	3.75	0.0109
Temperature (24 or 34°C)	1	609	341.36	<0.0001
Mass × stage	1	609	4.80	0.0289
Stage × population	3	609	2.47	0.0607
Stage × temperature	1	609	22.14	<0.0001
Population × temperature	3	609	8.03	<0.0001
Stage × population × temperature	3	609	14.58	<0.0001

The residual error was modelled with separate terms for each population and each temperature. The standard deviation of the mean for embryos from Colorado, New Jersey, and South Carolina was estimated to be 1.5, 1.4, and 1.4 times greater, respectively, than that for embryos from Arizona. The standard deviation at 34°C was 2.2 times greater than that at 24°C.

### Thermal limit of cardiac performance

**Figure 3:**
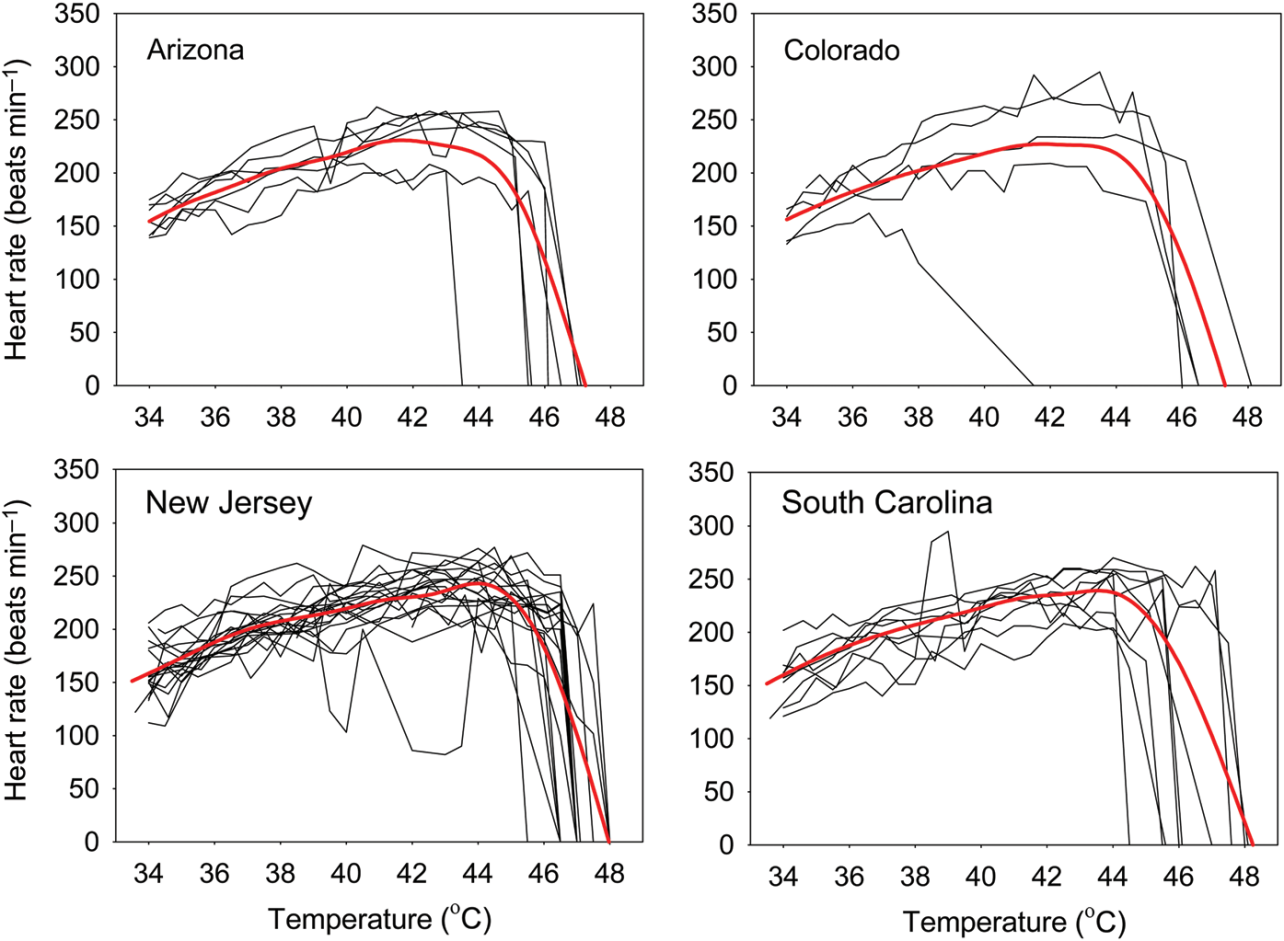
Heart rate increased during warming until embryos reached 41–44°C and dropped sharply between 44 and 47°C. Each black line depicts the trajectory of hearts rate for a single embryo. The red lines depict relationships estimated from the most likely general additive mixed model.

When embryos were warmed continuously, cardiac performance changed non-linearly in a manner commonly observed for physiological performances (Fig. 3). Warming from 34 to 41°C caused hearts to beat faster. Further warming caused heart rates to stabilize initially and then to drop precipitously. Cardiac arrest occurred at body temperatures ranging from to 41.5 to 48.1°C, but >93% of embryos succumbed at body temperatures >45°C. Older embryos in larger eggs reached slightly higher temperatures before cardiac arrest (Table 2). Although embryos from eastern populations reached higher heart rates (see Fig. 3), embryos from all populations underwent cardiac arrest at a similar median temperature, i.e. 46.5, 46.5, 47.0, and 46.5°C for embryos from Arizona, Colorado, New Jersey, and South Carolina, respectively (Table 3). The survival of heated eggs was extremely poor (7% hatching success) compared with that of eggs in the control group (51% hatching success). Survival was not influenced by water loss during warming, because the masses of eggs did not change appreciably during the warming experiment; mean masses before and after warming were 1.47 and 1.48 g, respectively (paired *t* = − 1.83, d.f. = 27, *P* = 0.08).

**Table 2:** Inferential statistics for the most likely model of heart rate during continuous warming

Effect	d.f.	*F*	*P*-value
*f*(*T*) for embryos from Arizona	7.79	42.73	<0.0001
Deviation from *f*(*T*) for embryos from Colorado	2.00	0.001	0.9993
Deviation from *f*(*T*) for embryos from New Jersey	5.82	6.47	<0.0001
Deviation from *f*(*T*) for embryos from South Carolina	4.60	5.10	0.0002

The generalized additive model describes heart rate as a function of temperature, or *f*(*T*), instead of using a fixed parameter to describe the effect of temperature. Additional functions were included to describe how heart rates of embryos from each population deviated from those of embryos from Arizona.

**Table 3:** Inferential statistics for the most likely model of the critical thermal maximum of heart rate

Effect	Effect d.f.	Error d.f.	*F*	*P*-value
Mass at oviposition (g)	1	34	17.55	0.1074
Stage of incubation (days)	1	34	11.97	0.8055
Population of origin (AZ, CO, NJ, SC)	3	34	0.54	0.0936
Mass × stage	1	34	9.96	0.0033

## Discussion

Heart rates of embryonic lizards either remained stable or decreased slightly during development. Generally, any developmental effect on heart rate was very small relative to the acute effect of temperature. The only pronounced decrease in heart rate during development occurred within embryos from Arizona; nevertheless, this pattern, which was observed only at 34°C, might have resulted from poor sampling of heart rates during later stages combined with the high variance of heart rate at 34°C (see Fig. 2). Our findings are in agreement with those of the only other study of developmental changes in heart rates of embryonic reptiles. In that study, heart rates of turtles at high temperature decreased during development, but heart rates at low temperature remained stable during development (Birchard and Reiber, 1996). In other embryonic vertebrates, heart rates increased, decreased, or remained stable during development (Tazawa, 2005; Brown *et al.*, 2012). Although heart rate often remains stable, the growth of embryos accelerates throughout most of development (Vleck and Vleck, 1980; Vleck and Hoyt, 1991). Consequently, the cardiac output needed for embryonic performance must come from an exponential increase of cardiac muscle, leading to greater stroke volume over time. The exponential increase in the stroke volume during the development of avian embryos accords with this interpretation (Tazawa, 2005).

Thermal sensitivities of embryonic heart rate in *S. undulatus* were similar to those of other species (Benfey and Bennett, 2009; Du and Shine, 2010; Lee and Lee, 2010). Du and colleagues (2010b, c) compared thermal sensitivities among several species of reptiles, including lizards, snakes, and turtles; heart rates increased by a factor of 1.5–2.6 with a warming of 10°C. In contrast, heart rates of avian embryos increase by 2- to 3-fold when warmed to the same extent (Nechaeva, 2011). In *S. undulatus*, heart rate barely increased by 2-fold over the range of 20–34°C (Du *et al.*, 2010a). Our measurements confirmed the low thermal sensitivity of reptilian heart rate, and also established that this thermal sensitivity remains stable throughout development (see Fig. 2). As with other physiological performances (Huey and Stevenson, 1979; Angilletta *et al.*, 2002b), the thermal sensitivity of heart rate depends on the range of temperatures; during continuous warming, heart rate initially increased rapidly but ultimately reached a maximum, resulting in a non-linear response to temperature over a broad thermal range (see Fig. 3).

Unlike previous experiments, we applied thermal stress until embryos failed to sustain cardiac performance. Heart rate decelerated rapidly at temperatures between 44 and 47°C (see Fig. 3). Contrary to our prediction, we did not find that the critical thermal maximum of cardiac performance was greater for embryos from warmer regions. This conservation of heat tolerance among populations suggests several hypotheses. First, selection caused by infrequent but extreme warming in all populations might have driven the critical thermal maximum to the highest possible level. If so, future warming would negatively impact the growth of populations without opportunities for adaptation to restore the mean fitness. Alternatively, mothers in different regions might choose nesting sites that never exceed the critical thermal maximum. In New Jersey, females construct nests at shallow depths (∼6 cm) in the most exposed soils they can find (Angilletta *et al.*, 2009). In more southerly environments, females can lay their eggs in deeper or shadier sites to compensate for the greater solar radiation. For nesting behaviour to compensate for changes in climate, females must differ genetically in their propensity to choose particular nesting sites (McGaugh *et al.*, 2010). Although nesting behaviour can compensate for temporal or spatial variation in environmental temperatures (Doody *et al.*, 2006; Telemeco *et al.*, 2009; Refsnider and Janzen, 2012), we currently know little about variation in the nesting behaviour of *S. undulatus* (Warner and Andrews, 2002; Angilletta *et al.*, 2009). Still, of the two hypotheses, the first one seems more plausible because nest temperatures in one of the coldest portions of the range sometimes exceed the critical thermal maximum (see below). Also, if adaptive nesting behaviour were a major factor, relaxed selection at the embryonic stage would enable critical thermal maxima to diverge among populations by genetic drift.

Interestingly, the temperatures that caused cardiac arrest in embryonic lizards exceeded temperatures that immobilized adult lizards from the same populations. Depending on its geographical origin, an adult cannot roll over at temperatures above 41–44°C (Angilletta *et al.*, 2002a; Ehrenberger, 2010). Unfortunately, we do not know the critical thermal maximum of heart rate in adults. In previous studies of *S. undulatus* (Dzialowski and O'Connor, 2001) and its congener *S. occidentalis* (Francis and Brooks, 1970), heart rates of adults increased linearly during warming, but warming did not exceed 35°C. In more distantly related species, heart rates of adult lizards increased linearly or almost linearly during warming to 40°C (Licht, 1965; Arad, 1995). Logically, the thermal limit of cardiac performance (and survival) must equal or exceed the thermal limit of locomotor performance. Nevertheless, the large difference between the thermal limits of embryonic heart rate and adult righting response suggest that embryos tolerate higher temperatures than adults do. Given that we pushed embryos to the point of cardiac arrest, we were unable to quantify any impact of sub-lethal stresses. In other words, exposure to temperatures slightly below the lethal limit might have impacted the phenotype or survival of embryos later in development.

**Figure 4:**
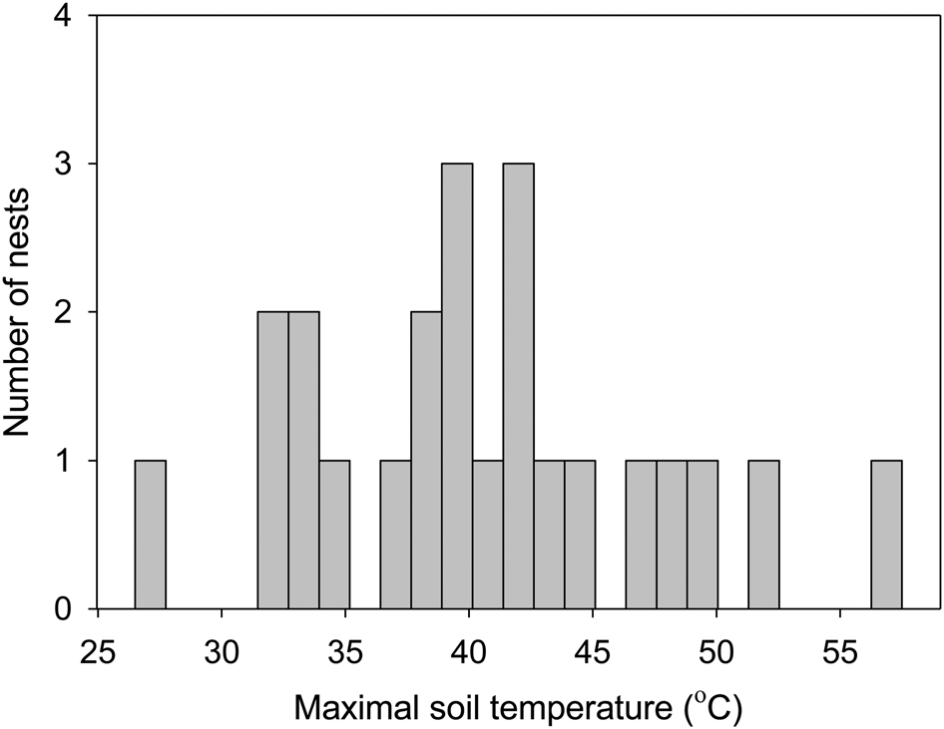
Embryos in natural nests experience temperatures that caused cardiac arrest in our experiment. The plot shows the distribution of maximal temperatures for nests in New Jersey (Angilletta *et al.*, 2009); nearly 22% of the nests exceeded the critical thermal maximum for this population (median = 47°C).

Studies of acute thermal stress provide insights needed to model the biological impacts of global warming. Having defined the temperatures that cause cardiac arrest, we can now infer whether temperatures in natural nests will remain within tolerable limits. In shallow soils, where lizards lay their eggs, brief exposures to extreme temperatures occur daily (Shine *et al.*, 2003; Angilletta *et al.*, 2009). For example, 22% of a sample of nests in New Jersey exceeded the critical thermal maximum for cardiac performance at least once (Fig. 4). This frequency of lethal events will increase if anthropogenic factors continue to drive global warming. To make matters worse, mortality can result not only from a single brief exposure to a high temperature but also from multiple exposures to lower temperatures. Thus, researchers should begin to consider the cumulative damage that occurs from sublethal heat stress. Surprisingly, we know even less about the sudden and cumulative impacts of extreme temperatures on embryos than we know about the ways in which embryos and their mothers thermoregulate (Du *et al.*, 2011; Schwarzkopf and Andrews, 2012; Shine, 2012). This gap in our knowledge exists because early research focused on constant temperatures and later research focused on non-lethal effects of fluctuating temperatures (Overall, 1994; Qualls and Shine, 1998; Shine and Downes, 1999; Andrews *et al.*, 2000; Oufiero and Angilletta, 2006). Clearly, this gap must be closed if we wish to know whether heat waves will threaten recruitment in populations.
